# Sex Differences in Wild Chimpanzee Behavior Emerge during Infancy

**DOI:** 10.1371/journal.pone.0099099

**Published:** 2014-06-09

**Authors:** Elizabeth V. Lonsdorf, A. Catherine Markham, Matthew R. Heintz, Karen E. Anderson, David J. Ciuk, Jane Goodall, Carson M. Murray

**Affiliations:** 1 Department of Psychology and Biological Foundations of Behavior Program, Franklin & Marshall College, Lancaster, Pennsylvania, United States of America; 2 Lester E. Fisher Center for the Study and Conservation of Apes, Lincoln Park Zoo, Chicago, Illinois, United States of America; 3 Center for the Advanced Study of Hominid Paleobiology, The George Washington University, Washington, DC, United States of America; 4 Committee on Evolutionary Biology, University of Chicago, Chicago, Illinois, United States of America; 5 Department of Government, Franklin & Marshall College, Lancaster, Pennsylvania, United States of America; 6 The Jane Goodall Institute, Vienna, Virginia, United States of America; CNR, Italy

## Abstract

The role of biological and social influences on sex differences in human child development is a persistent topic of discussion and debate. Given their many similarities to humans, chimpanzees are an important study species for understanding the biological and evolutionary roots of sex differences in human development. In this study, we present the most detailed analyses of wild chimpanzee infant development to date, encompassing data from 40 infants from the long-term study of chimpanzees at Gombe National Park, Tanzania. Our goal was to characterize age-related changes, from birth to five years of age, in the percent of observation time spent performing behaviors that represent important benchmarks in nutritional, motor, and social development, and to determine whether and in which behaviors sex differences occur. Sex differences were found for indicators of social behavior, motor development and spatial independence with males being more physically precocious and peaking in play earlier than females. These results demonstrate early sex differentiation that may reflect adult reproductive strategies. Our findings also resemble those found in humans, which suggests that biologically-based sex differences may have been present in the common ancestor and operated independently from the influences of modern sex-biased parental behavior and gender socialization.

## Introduction

Sex differences in behavior and developmental trajectories in human children are of great interest to researchers in a variety of fields of study. A persistent topic of discussion and debate is the relative contribution of biological versus social influences to such differences (reviewed in [1]) including how they may be driven by differential treatment by parents and teachers [2]. Given the potential effect of cultural and social influences on child development, non-human primates, and chimpanzees (*Pan troglodytes ssp*.) in particular, are an important study species for understanding the biological and evolutionary roots of sex differences in human development. Chimpanzees and humans have many genetic similarities [3] as well as similarities in physiology and growth [4]. Chimpanzees also share behavioral and developmental characteristics with humans, including a fission-fusion social system [5], [6], [7] and a relatively long period of nutritional and social dependency [8]. Moreover, the mother-infant relationship is of exceptional importance for developing chimpanzees given the lengthy period of dependence and lack of overt paternal care ([9], but see [10]).

Wild chimpanzee communities [11] are multi-male, multi-female and are characterized by a male dominance hierarchy in which philopatric males form the stable core of the community and defend a group territory [5]. Within these communities, adult male and female chimpanzees show distinct sex differences in behavior. These include differences in feeding and ranging patterns [12], such that females typically range and feed in small overlapping core areas [13] while males range more broadly throughout the territory. Additional sex differences in foraging include a female-bias towards gathering insects via tool use, and a male-bias towards hunting of vertebrate prey [14], [15]. In East African chimpanzees (*Pan troglodytes schweinfurthii*), there are distinct sex differences in sociality, such that adult females are significantly less gregarious than adult males, spending much of their time accompanied only by their dependent offspring (Gombe: [13], Kanyawara: [16], Mahale: [17]). As such, male-male dyads have stronger association indices than female-female dyads [18]. Male chimpanzees also participate in more direct physical aggression than females, both within communities during competition for dominance status and between communities during cooperative territorial defense [19], [20].

Previous research has described the general pattern of wild chimpanzee development. Physical contact with the mother characterizes most of the first two years of life [9]. Offspring are nutritionally dependent on their mother through infancy until weaning between the ages of 3 to 5, but remain behaviorally dependent (i.e. continually traveling and socializing with) through the juvenile years, until at least the age of 8 [11]. Only after the age of 10 years do most chimpanzees start to spend the majority of time away from their mother [8], [21] although it is not uncommon for adult chimpanzees (those over 12) to spend a significant amount of time with their mothers. In terms of physical growth, a long-term analysis of weight data from Gombe, Tanzania, showed that female chimpanzees are slightly lighter than males up to age 10, when adult dimorphic patterns begin to emerge and eventually result in a male/female body mass ratio of 1.25 [22].

Investigations of infant chimpanzee behavioral and social development and any sex differences therein have been more limited. Goodall [9] first outlined the typical stages of behavioral and social development of wild infant chimpanzees using a sample of four individuals through the first six months of life. Later stages of development were also described, although with small samples size at each stage. Clark [23] reported on mother-infant interactions surrounding weaning in six mother-infant pairs and Nicholson [24] compared the behavior of three captive mother-infant pairs with one wild pair. Subsequently, Plooij [25] and Ritj-Plooij and Plooij [26] focused on a cohort of six mother-infant pairs up to the age of 30 months but had limited ability to test for sex differences due to small sample sizes. Hiraiwa-Hasegawa [27] described age-related changes in nursing, and mode of transport (carrying versus independent traveling) in 16 wild mother-infant pairs from birth to 6 years of age, but did not find any sex differences in the development of those behaviors. Brent [28] investigated the effect of siblings on social relationships in infant chimpanzees from birth to 24 months and found suggestive, but not significant, differences in social behaviors such that males were slightly more social than females. Lonsdorf et al. [29] found sex differences in learning of a complex tool-use skill known as ‘termite-fishing’ such that infant female chimpanzees acquired the skill up to two years earlier than infant males. More recently, Lonsdorf et al. [30] documented sex differences in social behavior, showing that infant males have more social partners than infant females during the time period associated with their first independent forays into the social group (30–36 months).

In this study, we present the most detailed analyses of wild chimpanzee infant development to date, encompassing data from 40 infants from the long-term study of chimpanzees at Gombe National Park, Tanzania. Our goal was to characterize age-related changes, from birth to 5 years of age, in the percent of observation time spent performing behaviors that represent important benchmarks in nutritional, motor, and social development, and to determine whether and in which behaviors sex differences occur. We analyzed the percent of time (out of total observation time) each infant spent performing certain behaviors in each age block (described below). This ‘activity budget’ approach was preferred over an approach focusing on first appearance of behaviors given our interest in changes in behavior over time. The behaviors of interest represent a range of social and behavioral developmental indicators. We analyzed both suckling and eating solid food to investigate the development of nutritional independence. Time spent in social and solitary play, and social and self-grooming was analyzed to examine patterns of social development. We used the percent of time spent in three different behaviors to investigate physical/locomotor development: independent traveling (walking while their mother was also walking), riding ventrally on the mother’s belly (performed by very young infants) and riding dorsally on the mother’s back (performed by older infants) [9]. We also analyzed spatial independence by calculating the percent of time (out of total observation time) each infant spent in one of five estimated distance categories from the mother.

In addition to characterizing the average developmental trajectories of key behaviors for wild chimpanzees, our aim was to investigate the role of sex differences. We hypothesized that the well-documented sex differences in adult chimpanzee behavior would begin to emerge during infancy. Specifically, given the male bias towards gregariousness in adults [13], [16], [17], we hypothesized that male infants would spend more time in social behaviors, such as grooming and playing. Since physical interaction is a hallmark of adult male dominance behaviors [5], we hypothesized that male infants would show earlier physical and motor development, as measured by switching from riding on the belly to riding on the back, and travelling independently. In contrast, we made no prediction regarding sex differences for suckling or eating, given that previous studies of nursing in wild chimpanzees [27] reported no difference. Food acquisition is arguably of critical import to support the somatic growth of both males (for dominance interactions) and females (for reproduction).

## Materials and Methods

### Study Site

Gombe National Park is a small (35 km^2^) park, located on the western border of Tanzania (4.6667° S, 29.6333°E) and is home to three communities of chimpanzees. Our study focused on the Kasekela community which ranges in the center of the park and has been studied continuously since 1960. These chimpanzees are habituated to human observers, are individually recognized, and matrilineal kinship is known for as many as four generations. Historically, the Kasekela community has ranged in size from 38 to 64 individuals, with age-sex classes ranging from 6–14 adult males, 12–25 adult females, 6–14 subadult (<12 years of age) females and 7–15 subadult males.

### Ethics Statement

This study was completely observational in nature and the chimpanzees are well-habituated to human observation. Permission to conduct behavioral data collection at Gombe National Park was granted and approved by the relevant governing bodies in Tanzania: Tanzania National Parks, the Tanzanian Wildlife Research Institute, and the Tanzanian Commission for Science and Technology (permit # 2013-115-ER-2009-184).

### Behavioral Data Collection and Study Subjects

Detailed mother-infant behavioral data have been systematically collected on members of the Kasekela community since 1970. Focal follows on mother-infant pairs are conducted by two researchers who work in a team to record 1-minute point samples on the mother, her youngest offspring, and (when possible) observable older siblings. These point samples indicate proximity between the mother, infant and sibling (see distance categories below) and on-the-minute occurrence of various behaviors. For the analyses presented here, we focused on the following behaviors, as defined by the Gombe chimpanzee glossary (the Jane Goodall Institute, unpublished records):

1. Suckling – Infant’s mouth is in contact with the nipple.
2. Eating – Ingestion of solid food.
3. Solitary play – Includes swinging in a tree, somersaults, pirouettes, stamping and play-walk. May include an object (e.g. tossing, tumbling and self-tickle with a detached object).
4. Social play – Non-aggressive interaction between two or more individuals that include one or more of the following: tickling, wrestling, chasing, kicking, rubbing, thrusting, biting, or pulling. May incorporate an object (e.g. tugging of sticks back and forth).
5. Social groom – Parting of another individual’s hair with hands, fingers and/or lips and removal of debris or ectoparasites.
6. Self-groom – As above but performed on self.
7. Travel – Continuous movement from one point to another.
8. Riding ventrally – The infant is transported as it clings to the mother’s belly, gripping hair between flexed fingers and toes.
9. Riding dorsally – The infant is transported as it lays or sits on the mother’s back.

For these analyses, we focused on infancy, from birth to 5 years of age. For statistical analyses, this time period was broken into age blocks that were comprised of 60 days during the first year (to capture the more rapid changes that occur), and 90 days thereafter. Based on these time blocks we set an inclusion criterion for individual infants of at least 10 hours of data for each 60-day block for the first year, and 15 hours of data for each 90-day block thereafter. Applying these criteria, we included a total of 15 female and 25 male offspring, with mean (+/− SD) observation minutes per block of 1474 (+/−636) and 1343 (+/−408), respectively. These data were collected from 1988 to 2011.

### Developmental and Spatial Independence Metrics

We analyzed the percent of time (out of total observation time) each infant spent performing certain behaviors in each age block (described above). Minutes during which the infant was out-of-view were excluded. To examine spatial independence, we analyzed the percent of time (out of total observation time) each infant spent in one of five estimated distance categories: (1) contact, (2) within arm’s reach, (3) arms reach to 5 m, (4) 5 to 15 m, (5) greater than 15 m. We collapsed distance categories into a single continuous variable by assigning the midpoint of each category to each point sample and computed a weighted average. The assigned midpoints were 0m, 0.5 m, 3 m, 10 m, and 15 m respectively. Since distance category 5 does not have a midpoint (it is not bounded by an upper limit on distance), we used 15 m so as to compute the most conservative estimates. We then analyzed this continuous distance variable using GLMMs as described below.

### Statistical Analyses

Our analyses focused on how time spent in different behaviors changed with age, and whether these changes differed by infant sex. For behaviors that could be modeled using linear effects we used Statistical Analysis Software version 9.2 (SAS; Cary, NC). Because there were multiple and unbalanced numbers of data points from each offspring, we used a mixed model format (PROC MIXED) in order to include a random effect for each infant. For behaviors that could not be modeled using linear effects, we used generalized additive mixed models (GAMMs), using the mgcv package in R (version 3.0.1, R Core Development Team 2013). These models fit smooth functions to non-linear data and also allow the inclusion of random effects for repeated measures on the same subject. A generalized additive model (GAM) is a generalized linear model with a linear predictor involving a sum of smooth functions of covariates. A generalized additive mixed model (GAMM) is an extension of the GAM that allows the inclusion of random effects for repeated measures on the same subject. Just as a GAM is an extension of a GLM, a GAMM is an extension of a GLMM [31].

## Results

For ease of visualization and interpretation, Table 1 and all figures display summary statistics for six-month age blocks. We conducted statistical analyses on narrower time blocks as described above. Hereafter, when referring to patterns at particular ages, we will use the age at the start of the block to refer to a six month time period (e.g. ‘age 2′ refers to the six month time block from age 2 to 2.5 years).

**Table 1:** Percent of time spent performing the indicated behavior, summarized for 6-month intervals.

Age (yrs)	# Inds(M, F)	*Travel	*Ride back	*Ride belly	*Social play	Solitary play	Groom other	Self groom	Eat
0	22 (12,10)	**0.00** (0.00)	**0.04** (0.02)	**11.90** (1.23)	**0.72** (0.24)	**3.49** (1.39)	**0.01** (0.00)	**0.00** (0.00)	**0.23** (0.10)
0.5	21 (13,8)	**0.20** (0.10)	**2.91** (0.66)	**9.13** (1.06)	**4.69** (0.98)	**16.17** (1.87)	**0.15** (0.06)	**0.06** (0.03)	**5.33** (1.01)
1	16 (10, 6)	**0.56** (0.21)	**7.25** (0.96)	**5.99** (0.92)	**9.53** (1.06)	**21.78** (1.95)	**0.11** (0.03)	**0.01** (0.01)	**6.67** (0.86)
1.5	15 (9, 6)	**1.52** (0.28)	**8.58** (0.81)	**3.06** (0.40)	**11.41** (1.48)	**18.37** (2.30)	**0.35** (0.14)	**0.09** (0.04)	**21.95** (3.12)
2	11 (8, 3)	**2.29** (0.50)	**8.93** (0.59)	**2.08** (0.47)	**15.16** (2.08)	**16.16** (2.74)	**0.27** (0.12)	**0.15** (0.08)	**21.64** (2.54)
2.5	15 (9, 6)	**3.45** (0.58)	**7.50** (0.97)	**1.64** (0.64)	**12.39** (1.84)	**14.76** (2.61)	**0.75** (0.28)	**0.41** (0.20)	**29.74** (2.82)
3	11 (7, 4)	**4.96** (1.23)	**4.98** (1.03)	**0.65** (0.17)	**13.70** (2.68)	**9.12** (1.62)	**0.83** (0.21)	**0.22** (0.12)	**32.45** (5.63)
3.5	15 (9, 6)	**5.65** (0.89)	**3.36** (0.64)	**1.04** (0.31)	**10.10** (1.64)	**5.83** (1.30)	**2.23** (0.88)	**0.43** (0.16)	**36.14** (3.01)
4	9 (3, 6)	**4.88** (1.30)	**5.13** (1.44)	**0.96** (0.41)	**8.86** (3.06)	**4.60** (1.34)	**2.68** (0.93)	**0.71** (0.40)	**34.43** (3.96)
4.5	9 (5, 4)	**8.72** (2.02)	**1.25** (0.51)	**0.16** (0.06)	**7.22** (1.72)	**2.96** (0.82)	**3.07** (1.27)	**0.51** (0.25)	**49.09** (4.13)

Age at the start of the interval is given in years. Total number of individuals is listed for each age class and by sex. Values given are mean (se).

*Behaviors in which sex differences in developmental trajectories are present.

### Suckling and Eating

Suckling did not show a significant change with age or sex, with mean percent of time observed suckling ranging from 2–3.71%. The percent of observation time spent dedicated to eating solid food increased significantly with age (F_1,34_ = 278.91, p<0.0001), from an average of 0% of time in the first six months to 49.09% of observation time at 4.5 years (see Table 1). The effect of sex was not significant.

### Social and Solitary Play

**Figure 1:**
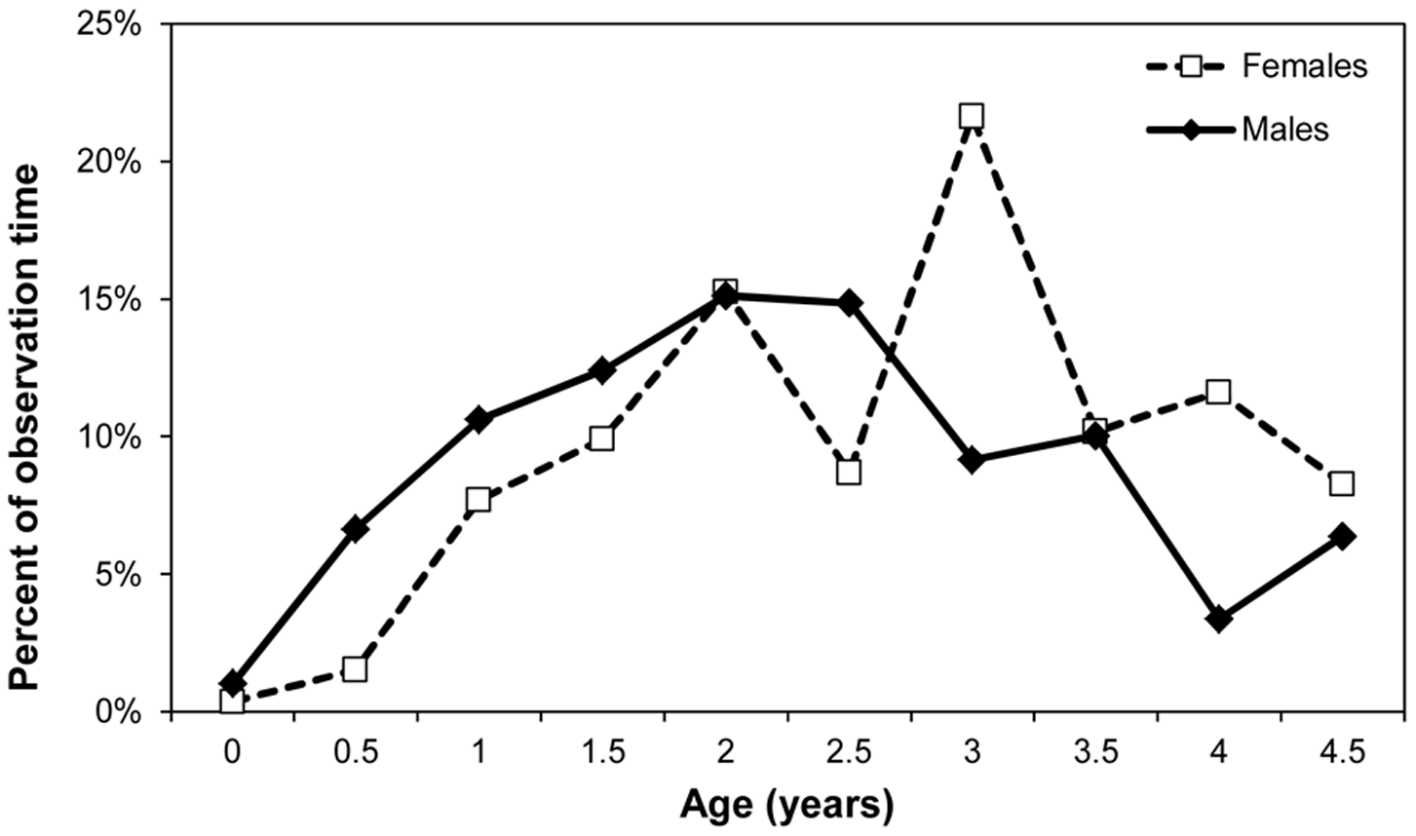
Mean percent of observation time spent in social play for male and female infants.

**Figure 2:**
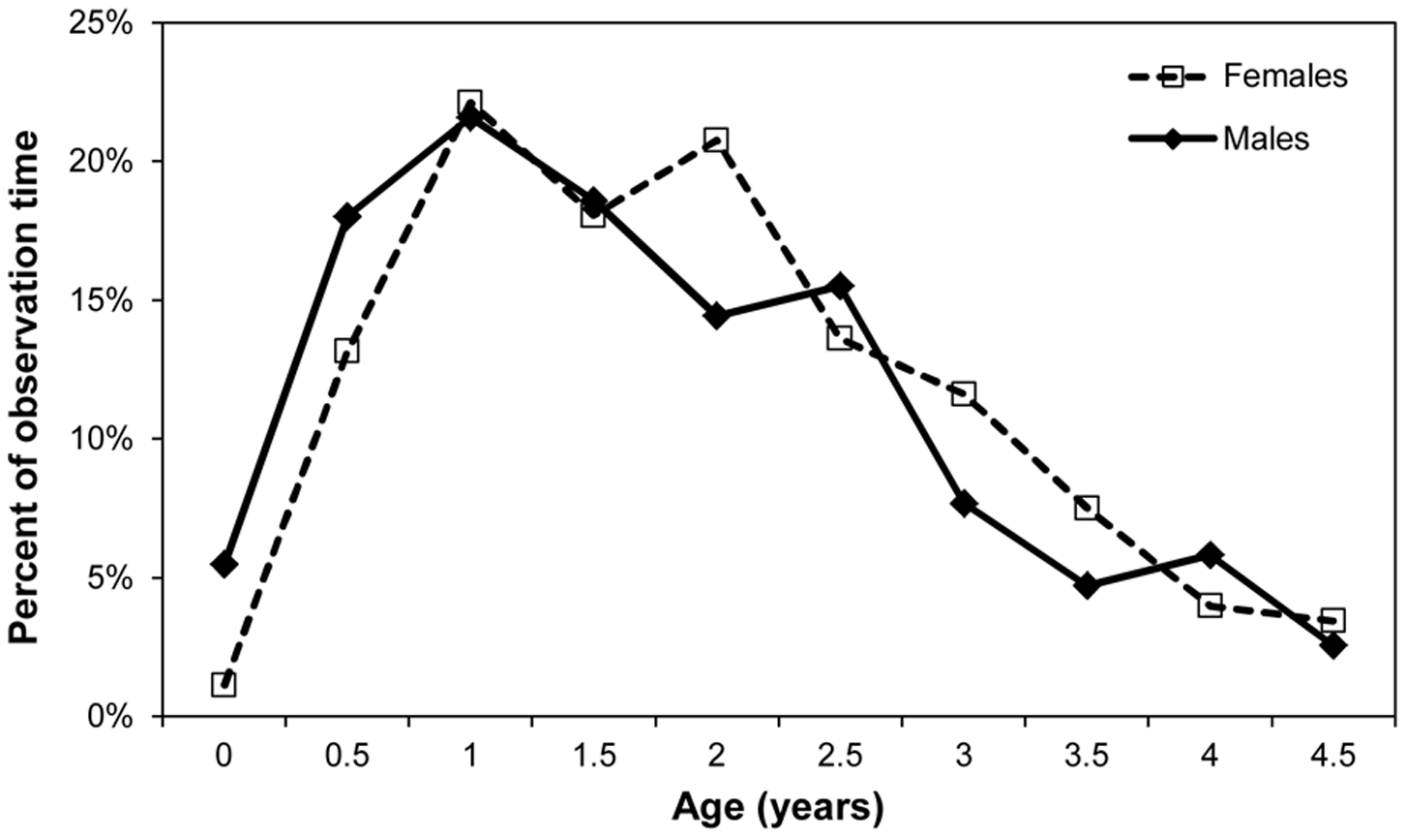
Mean percent of observation time spent in solitary play for male and female infants.

When fitted with GAMMs, the best fit model for percent time spent in social play was one in which separate smooth curves were fit to age for each sex, which is analogous to an age by sex interaction in a general linear model (smooth over age for both males and females p<0.001, adjusted R^2^ = 35.3%). Table 1 shows that overall, social play peaks during infancy at around age 2, with 15.16% percent of observation time on average spent playing with others. However, Figure 1 shows that male infants have increased amounts of social play when compared to female infants at earlier ages and that female infants’ peak in time dedicated to social play is later. Solitary play shows no such sex difference. The best fit GAMM model is a smooth over age only (p<0.001, adjusted R^2^ = 44%). Figure 2 shows that solitary play peaks for both males and females in the age class beginning at 1 year at 21.78% and reduces to below 5% of observation time by age 4.

### Social and Self-grooming

We used GLMMs to analyze grooming behaviors. The percent of observation time dedicated to social grooming increased significantly with age (F_1,34_ = 30.8, p<0.0001), from an average of 0% of time in the first six months to 3.07% of observation time at age 4.5 years (see Table 1). Self-grooming occurred at very low levels overall, but still increased significantly with age (F_1,34_ = 19.91, p<0.0001) from an average of 0% of time in the first six months to an average of 0.51% of time at age 4.5 years. The effect of sex was not significant.

### Riding and Travel

**Figure 3:**
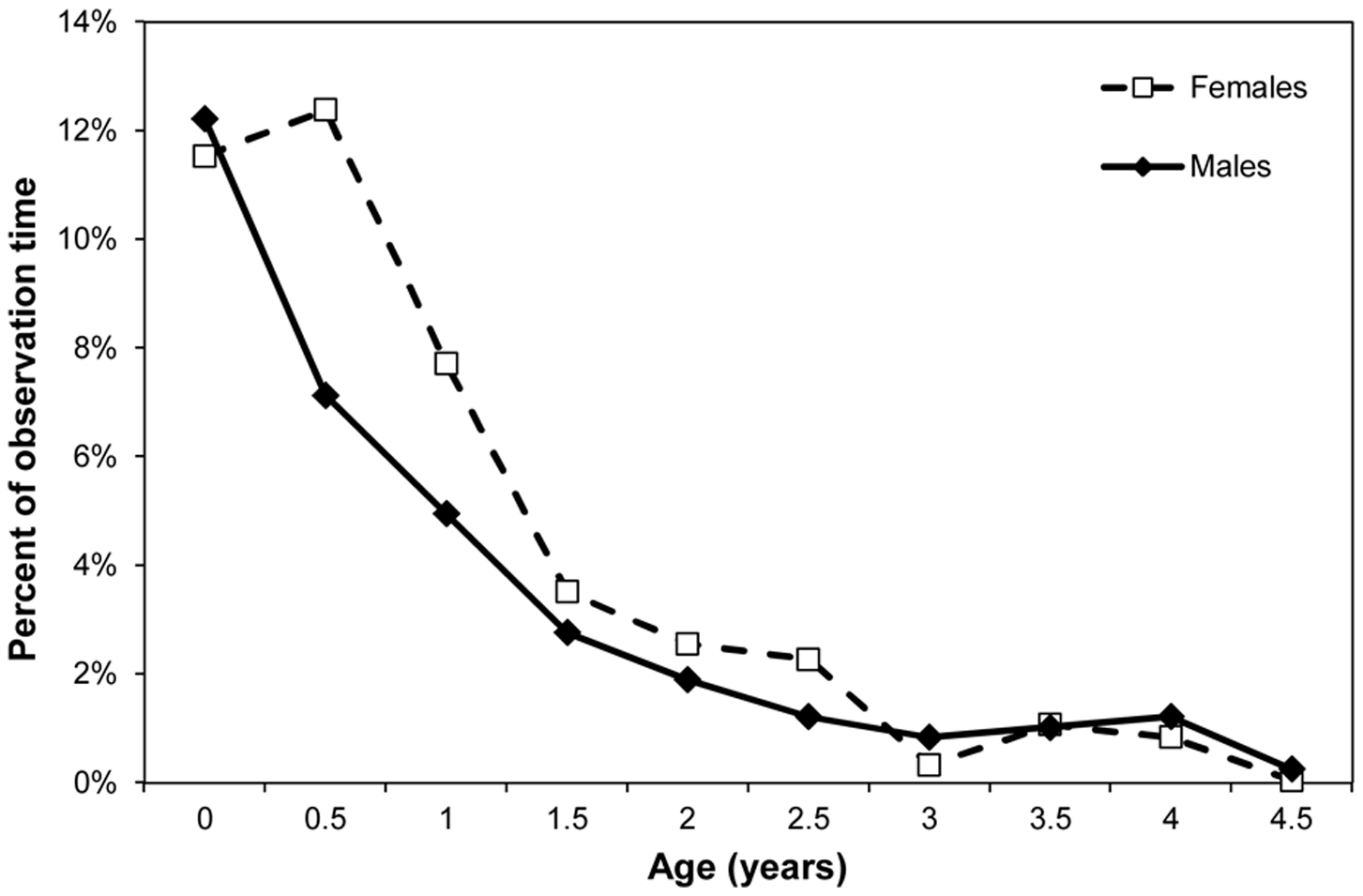
Mean percent of observation time spent riding ventrally for male and female infants.

**Figure 4:**
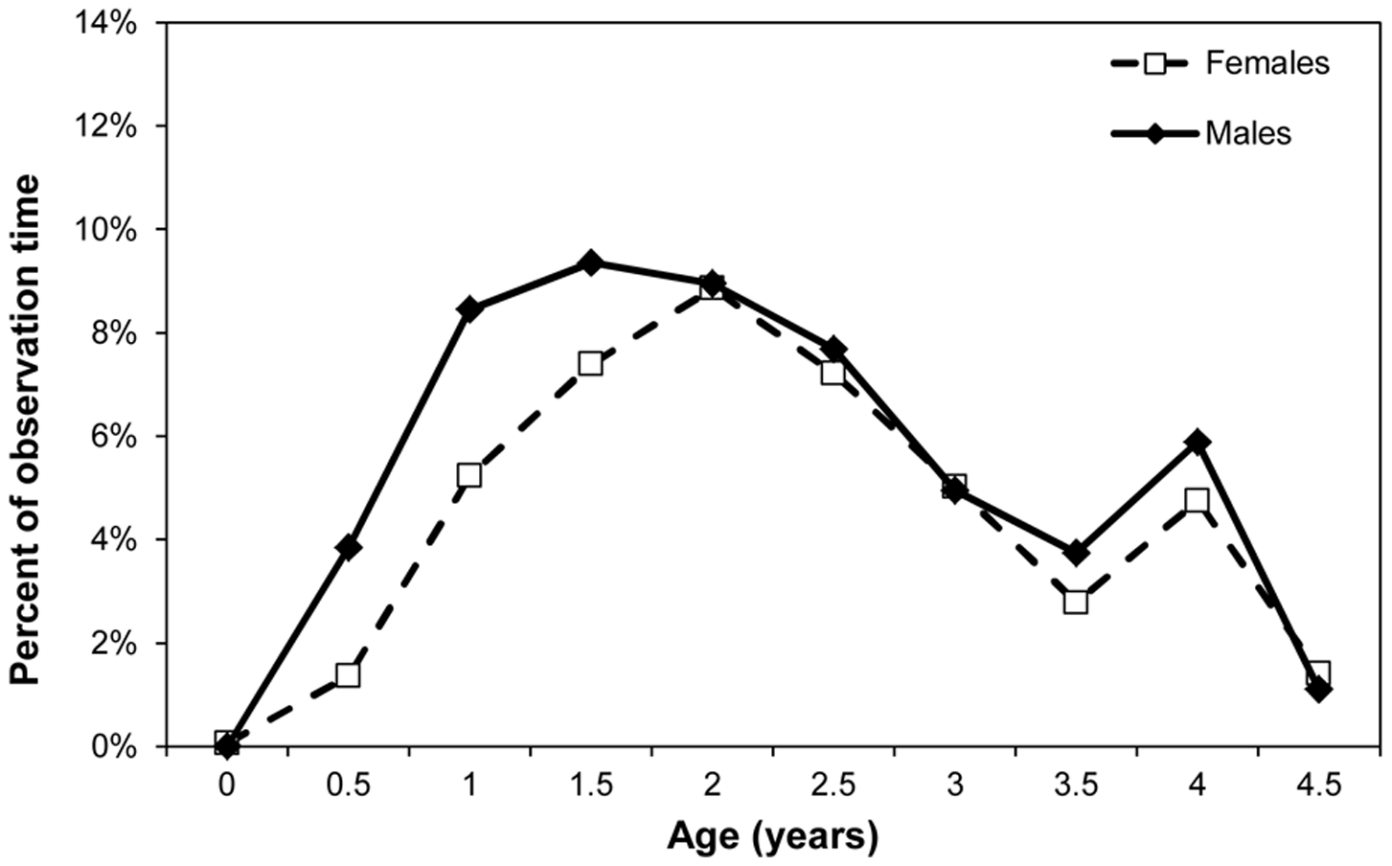
Mean percent of observation time spent riding dorsally for male and female infants.

**Figure 5:**
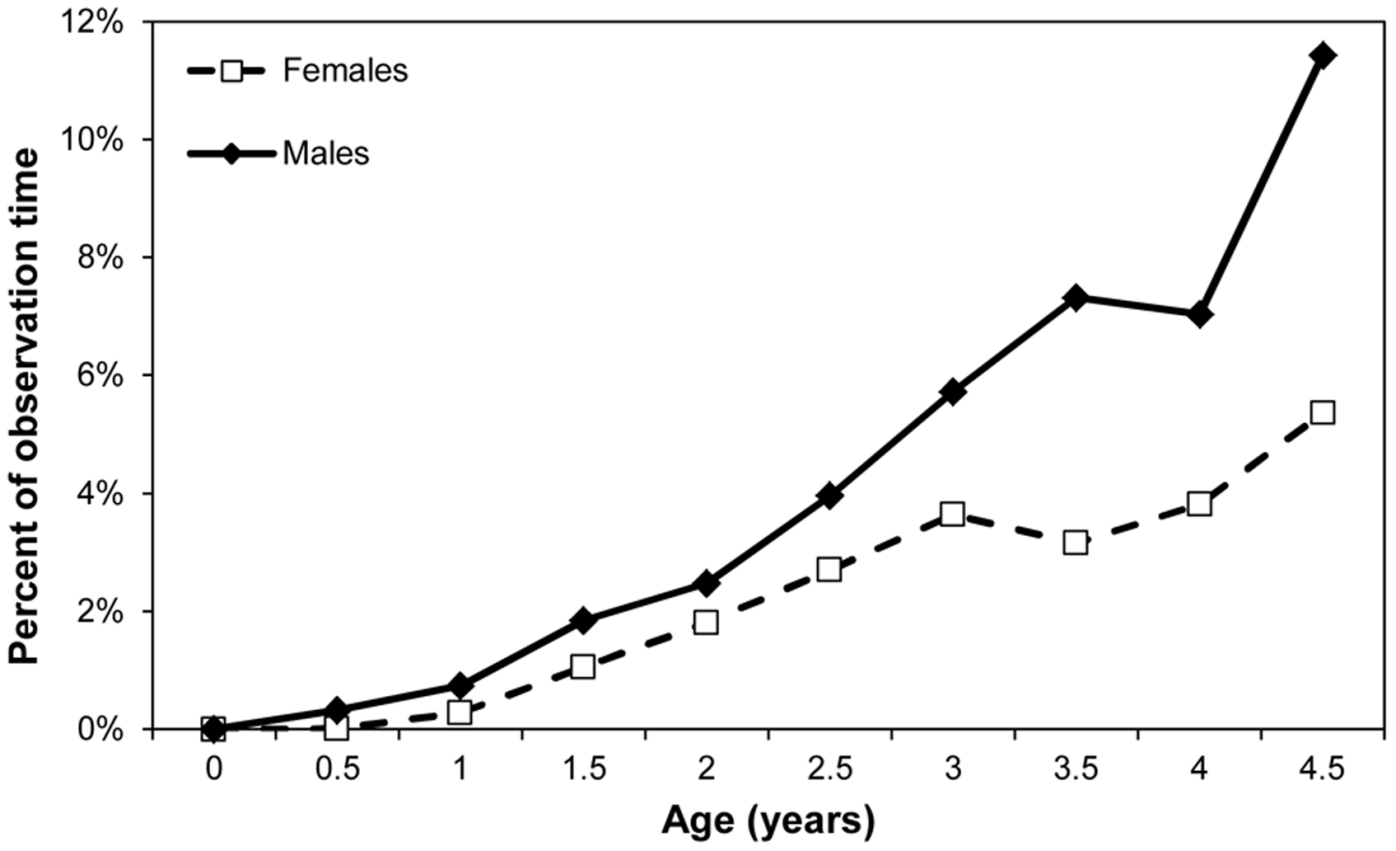
Mean percent of observation time spent traveling independently for male and female infants.

We used GLMMs to model the percent time spent riding ventrally. Age was significant (F_1,34_ = 233.2, p<0.0001), while sex was marginally significant (F_1, 148_ = 3.86, p = 0.0512). We used GAMMs to model the percent time spent riding dorsally. The best fit model for percent time spent riding on the back included a smooth over age (p<0.001) and a fixed effect for sex (p = 0.0843, adjusted R^2^ = 50.3%). Figures 3 and 4 show the sex difference. Male infants reduce the time they spent riding ventrally and increase the time spent riding dorsally earlier than female infants. We used GLMMs to model percent of time spent traveling independently, and both age (F_1,34_ = 109.34, p<0.0001) and the age by sex interaction (F_1,148_ = 10.52, p = 0.0015) were significant. Figure 5 shows that male infants begin to travel independently earlier than females, and do so more at later ages.

### Spatial Independence

**Figure 6:**
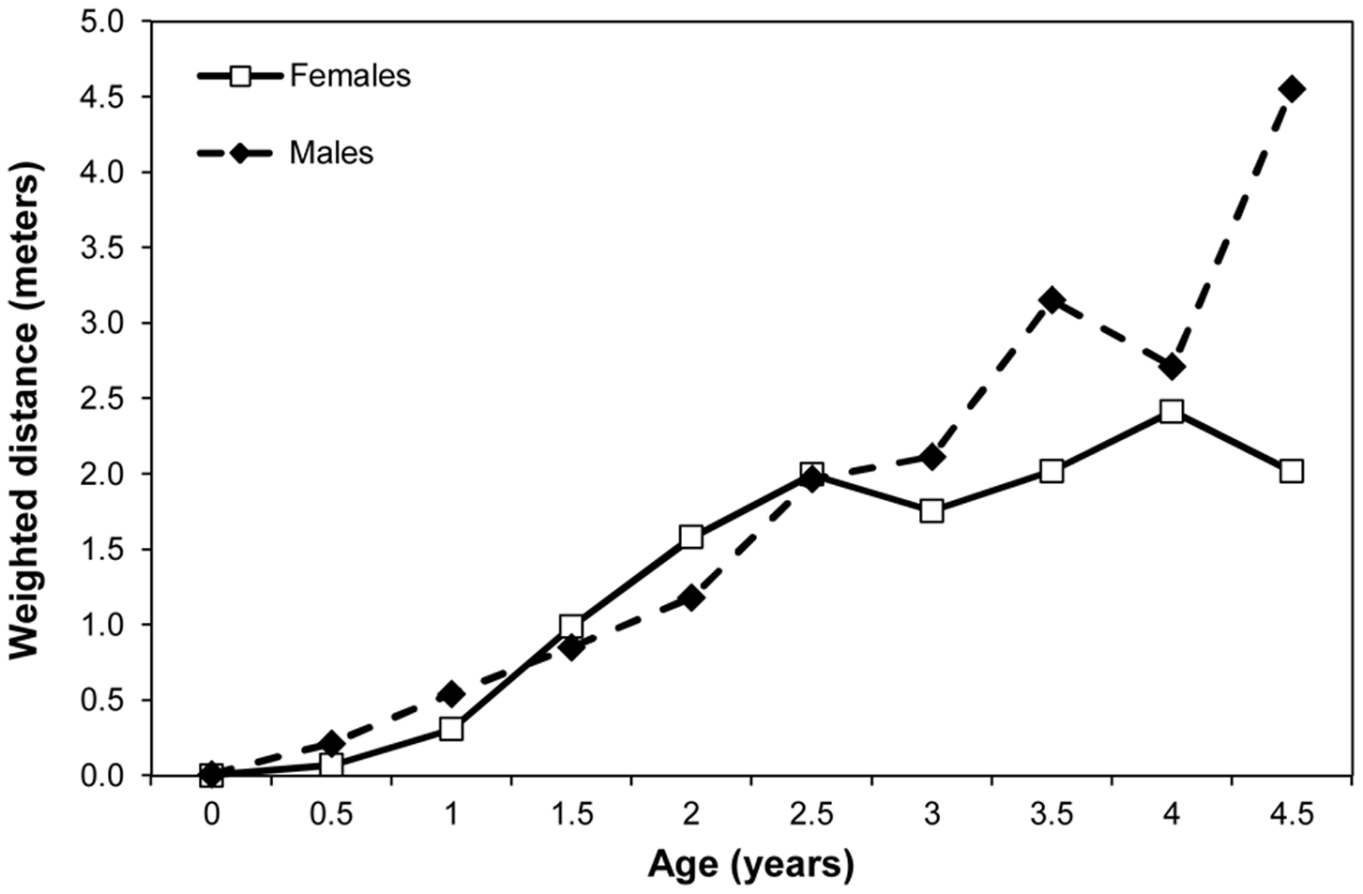
Mean weighted distance measure (in meters) by age of female and male infants.

We used GLMMs to analyze the effect of age and sex on a weighted estimate of distance between mother and offspring. Both age (F_1,33_ = 142.75, p<0.0001) and the age by sex interaction (F_1,152_ = 5.96, p = 0.0158) were significant. Figure 6 shows that male infants begin to show increased distance from their mothers at age 3 and continue to be at farther distances than female infants thereafter.

## Discussion

This study provides the most complete description of wild infant chimpanzee developmental trajectories to date and offers an important point of comparison for other studies of human and non-human primate development. Sex differences between male and female chimpanzee infants were apparent in some, but not all, of the behaviors we analyzed. For example, no sex difference was apparent for suckling which also did not decrease predictably with age. While perhaps counterintuitive, this result complements that of suckling percentages found at other sites (Kanyawara: [32], Mahale: [27]) in showing that chimpanzee infants continue suckling well after the introduction of solid food into the diet. This suggests that suckling may fulfill a combination of nutritional and social (comfort-related) needs. Additionally, the relationship between suckling and actual volume of milk intake is currently unknown for wild chimpanzees and recent research in other primate species has found that both milk volume and milk composition vary according to sex of the infant [33], [34]. As such, the nutritional importance of suckling requires further research. Similarly, no sex differences were found in the amount of time dedicated to eating solid food, which increased predictably with age to approximately 50% of observable time by age five. Socioecological theory predicts [12] and long-term studies have documented that female reproductive success is limited by access to food [13], [16], [35] while male reproductive success is limited by access to females [36], [37]. Therefore, food intake during infancy may be equally important to support somatic growth for developing male offspring to support male-male competition, and female offspring to support earlier age of first reproduction. Future studies should attempt more detailed nutritional analyses to determine whether specific differences exist in the types of food consumed by male and female infants or if differences emerge later in development.

While sex differences in play are fairly widespread in juvenile primates (reviewed in [38]), less is known about sex differences in infancy. In blue monkeys [39] and olive baboons [40], male infants play for longer periods of time than female infants, while in rhesus macaques, male infants show more rough-and-tumble play, chasing play, and play initiation [41]. Our data suggest that play is an important part of chimpanzee infancy given that together, solitary and social play comprise upwards of 30% of an infant’s observation time in some years (see Table 1). Sex differences were apparent in social, but not solitary play, with males dedicating more time at earlier ages to social play and females peaking in percent of time engaged in social play later. As described elsewhere, male infants aged 30–36 months show a more diverse set of social partners [30] and are particularly biased towards interacting with adult males. These complementary lines of evidence may reflect the relative importance of socialization for young males given the importance of social dominance in adulthood. Both males and females show a decline in amount of play in late infancy, which may be related to weaning conflict. Further research on play rates during juvenility will allow us to better understand the relationship between play during development and adult sex-specific social behavior.

Our results cast an important, comparative light on sex differences in social development and play in human children, which have been documented through a variety of metrics. Girls tend to outperform boys in the realm of prosocial behavior, even at very young ages. For example, female neonates prefer looking at pictures of a face, while male neonates prefer looking at pictures of a mobile [42], suggesting an innate sex difference in preferences for social versus mechanical objects. In addition, girls establish and maintain eye contact more than boys [43] and engage in more play parenting [44]. However, play parenting differences become more marked in juvenility and beyond [45] and are likely heavily influenced by parents, who more often assign child-care roles to girls [46]. In studies of peer social play, researchers have found a male-bias towards larger amounts of play [47] and more rough and tumble play by age 3 [48]; these sex differences continue to increase through juvenility and beyond. Our full dataset did not differentiate play types, so future research is needed to explore sex differences in play type and partners. Such data will allow a more complete comparison between human and non-human primate play trajectories.

In contrast, self- and social grooming accounts for exceptionally small amounts of a chimpanzee infant’s observed time and there were no apparent sex differences. Sex differences in grooming have been found in other primate infants. In rhesus macaques [49] female infants groom others more than males in the first year of life, and grooming continues to develop along sex-specific social lines through puberty and early adulthood. Human girls also engage in more grooming than boys [47]. As pointed out by Roney & Maestripieri [49], sex differences in grooming by young primates are adapted to and reflect the particular social structure of the species. For chimpanzees, grooming likely becomes more important during the juvenile and adolescent years, when individuals are beginning to ascend their respective dominance hierarchies and sex-specific grooming relationships begin to differentiate [8].

Precise physical and motor developmental changes are hard to measure in wild chimpanzees, although advances have been made in investigating skeletal remains [50] and using photographic techniques [32]. Here we used changes in riding posture and independent traveling as proxies for motor development. Males switch from riding ventrally (the more immature mode of travel) to riding dorsally earlier than females. Male infants also begin traveling independently earlier and spend more time traveling independently than females overall. This is in contrast to the earlier findings of Hiraiwa-Hasegawa [27], however, our larger dataset with more individuals per age and newer statistical techniques may have allowed for more detailed analyses. These data are linked with and mirrored in the results for distance, which is a measure of increasing spatial independence. Male infants are at farther distance from their mother by age 3 and remain at greater distances than females up to age 5. Pusey [21] found similar differences in proximity in juveniles and adolescents; male juveniles spent less time than females within 15m of their mothers and also spent greater amounts of time ‘leading’ their mothers during travel. Thus, faster motor development in males as measured by riding position is followed by earlier and more independent traveling and greater spatial independence from their mothers. These differences in infancy then persist through the juvenile and adolescent stages of development.

Taken together, these results complement findings from the human literature on sex differences in activity levels and independence from the mother. For example, infant boys have increased motor activity level compared to infant girls [51] and showed more leg activity both ante- and neonatally than girls [52]. Sex differences in achievement of motor milestones by age 24 months have not been found in human children [53]. However, by preschool and grade school, males outperform females on the majority of gross motor tasks studied [54]. With regards to earlier independent traveling and spatial independence, our results parallel those found in human studies in that boys spend more time away from their mothers than girls [47] and engage in more risk-taking behaviors [55].

In sum, we have characterized the average developmental trajectories of several key behaviors in a large sample of wild chimpanzees. We found sex differences in social play, motor development and spatial independence that are consistent with adult sex-specific social roles in chimpanzees and parallel similar patterns found in humans. Further detailed investigations of types of play and play partner preferences are warranted. We did not find any sex differences for suckling or eating, but future studies would be useful to gain more precise measurements of nutrient intake. We also did not find any sex differences in grooming, which may become more apparent later in development. Our similar, albeit not precisely comparable, findings in wild chimpanzees suggests that some biologically-based sex differences in behavior may have been present in the common ancestor of chimpanzees and humans, and operated independently from the influences of modern sex-biased parental behavior and gender socialization. Future research will allow us to understand whether variation from these trajectories are influenced by particular maternal characteristics (rank, parity, and/or maternal style) and/or result in differing outcomes for offspring.
